# Engineered Microparticles for Treatment of Murine Brain Metastasis by Reprograming Tumor Microenvironment and Inhibiting MAPK Pathway

**DOI:** 10.1002/advs.202206212

**Published:** 2023-01-25

**Authors:** Lisen Lu, Huaduan Zi, Jie Zhou, Jing Huang, Zihan Deng, Zijian Tang, Li Li, Xiujuan Shi, Pui‐Chi Lo, Jonathan F. Lovell, Deqiang Deng, Chao Wan, Honglin Jin

**Affiliations:** ^1^ College of Biomedicine and Health and College of Life Science and Technology Huazhong Agricultural University Wuhan 430070 P. R. China; ^2^ Cancer Center Union Hospital Tongji Medical College Huazhong University of Science and Technology Wuhan 430022 P. R. China; ^3^ Beijing Institute of Clinical Research Beijing Friendship Hospital Capital Medical University Beijing 100050 P. R. China; ^4^ Department of Biomedical Sciences City University of Hong Kong Tat Chee Avenue Kowloon Hong Kong HKG P. R. China; ^5^ Department of Biomedical Engineering University at Buffalo State University of New York Buffalo NY 14260 USA

**Keywords:** blood‐brain barrier, cancer immunotherapy, extracellular vesicles, macrophages polarization, USP7

## Abstract

Brain metastases (BRM) are common in advanced lung cancer. However, their treatment is challenging due to the blood‐brain barrier (BBB) and the immunosuppressive tumor microenvironment (ITME). Microparticles (MPs), a type of extracellular vesicle, can serve as biocompatible drug delivery vehicles that can be further modulated with genetic engineering techniques. MPs prepared from cells induced with different insults are compared and it is found that radiation‐treated cell‐released microparticles (RMPs) achieve optimal targeting and macrophage activation. The enzyme ubiquitin‐specific protease 7 (USP7), which simultaneously regulates tumor growth and reprograms M2 macrophages (M2Φ), is found to be expressed in BRM. Engineered RMPs are then constructed that comprise: 1) the RMP carrier that targets and reprograms M2Φ; 2) a genetically expressed SR‐B1‐targeting peptide for improved BBB permeability; and 3) a USP7 inhibitor to kill tumor cells and reprogram M2Φ. These RMPs successfully cross the BBB and target M2Φ in vitro and in vivo in mice, effectively reprogramming M2Φ and improving survival in a murine BRM model. Therapeutic effects are further augmented when combined with immune checkpoint blockade. This study provides proof‐of‐concept for the use of genetically engineered MPs for the treatment of BRM.

## Graphical abstract

Microparticles derived from Lewis Lung Carcinoma cells treated with radiation have the strongest targeting and reprogramming effects on macrophage. When loaded with ubiquitin‐specific protease 7 inhibitor and R4F peptides, the particles can actively cross the Blood Brain Barrier and reprogram macrophage in the tumor microenvironment, effectively prolong the survival rate of murine lung cancer brain metastases model through combined immunotherapy.

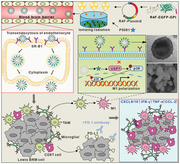

## 1 Introduction

The incidence of lung cancer is second only to breast cancer, and the mortality rate ranks first among all cancers.^[^ ^1^ ^]^ Nearly 80% of lung cancer patients develop multiple brain metastases in the late stage.^[^ ^2^ ^]^ The main clinical strategies for the treatment of lung cancer brain metastases (BRM) are radiotherapy and chemotherapy.^[^ ^3^ ^]^ However, radiotherapy often can cause irreversible damage to the nervous system, meanwhile the blood‐brain barrier (BBB) prevents the effective infiltration of chemotherapy drugs into target sites.^[^ ^4^ ^]^ The rapid development of immunotherapy and the discovery of lymphatic vessels in the brain provide a new therapeutic strategy for BRM.^[^ ^5^ ^]^ However, the immunosuppressive tumor microenvironment (ITME) limits the efficacy of immunotherapy in patients with BRM.^[^ ^6^ ^]^ Therefore, there is a need to develop new therapeutic strategies that can reverse the ITME of BRM.

Effective brain‐targeted delivery systems are a prerequisite for promoting strong drug infiltration into the central nervous system (CNS) and improving the ITME of brain tumors. Autologous tumor cell‐derived microparticles (MPs, containing DNA, RNA, proteins and lipids from the cell, primarily microvesicles herein) have attracted interest as they are safe, are easily modified, and possess large drug loading capacity and specific targeting characteristics.^[^ ^7^ ^]^ Therefore, MPs are well‐suited as drug vehicles for personalized cancer treatments. MPs can deliver tumor cells antigens or other autologous cell‐associated antigens to DCs and induce CD8^+^ T‐cell‐dependent anti‐tumor responses or immunosuppressive responses in vivo.^[^ ^8^, ^9^ ^]^ MPs produced by ultraviolet (UV)‐irradiated tumors combined with methotrexate have been tested in patients with extrahepatic cholangiocarcinoma or malignant pleural effusion and showed good therapeutic effect.^[^ ^10^, ^11^, ^12^ ^]^

MPs may vary in function depending on the conditions under which they were obtained; for example, MPs secreted from tumors under physiological conditions can promote tumor progress,^[^ ^13^ ^]^ while MPs produced by tumors exposed to radiotherapy, hyperthermia, and UV or acid‐base stimulation have certain immune activation functions.^[^ ^12^, ^13^, ^14^, ^15^ ^]^ However, the extent of the anti‐tumor functions of MPs produced by different treatments remains to be explored. Additionally, MPs have some innate function across the BBB, possibly due to the expression of integrins and tetraspanins and some ligands for specific receptors on the receiving cells. This targeting or penetration effect may be further potentiated through surface decoration with a variety of ligands, such as transferrin, insulin, apolipoprotein E, angiopep‐2, and antibodies.^[^ ^16^ ^]^ However, MPs would not be sufficient to effectively reverse the ITME in brain. Therefore, it is necessary to combine a suitable MP platform with other strategies to target and treat BRM.

Tumor‐associated macrophages (TAMs) release immunosuppressive factors such as IL‐10 and TGF‐beta, induce the exhaustion of effector T‐cells, and promote the formation of myeloid‐derived suppressor cells (MDSCs). Therefore, TAMs are one of the main contributors to the ITME, accounting for nearly 50% of the infiltrating immune cells in the ITME of BRM.^[^ ^17^ ^]^ Moreover, TAMs possess relatively conserved gene stability compared with heterogeneous tumor cells, making them a key therapeutic target for reversing the ITME. Methods to reduce M2 macrophages (M2Ф) include knockdown approaches involving CSF‐1R siRNA and related antibodies,^[^ ^18^ ^]^ and autophagy intervention with nanomaterials.^[^ ^19^ ^]^ To reprogram M2Ф, several nanocarriers have been constructed to deliver mRNA encoding M1‐polarizing transcription factors to CD206‐expressing M2Ф.^[^ ^20^ ^]^ Additionally, some studies have reported that exosomes derived from M1 macrophages (M1Ф) can be extracted for use in the reprogramming of M2Ф by activating the NF‐*κ*B signaling pathway or transferring M1Ф‐associated transcription factors.^[^ ^21^ ^]^ Although these strategies can promote the polarization of M2Ф, a more appealing approach would be the identification of a dual target that can simultaneously regulate both the tumor and M2Ф. Previously, we have shown that the expression of the de‐ubiquitination enzyme USP7, an oncogene in the subcutaneous Lewis tumor model, is much higher in M2Ф than in M1Ф. Inhibition of USP7 can effectively reverse ITME in subcutaneous tumor models of lung cancer.^[^ ^22^ ^]^ However, the role of USP7 in BRM has not been reported.

**Figure 1:**
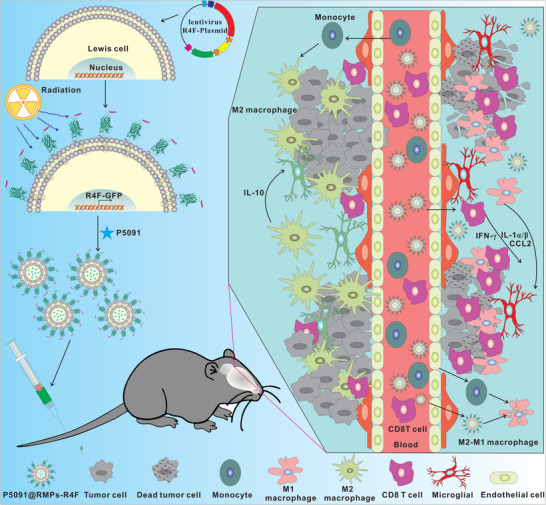
This study demonstrates an intravenous genetically engineered microparticle loaded with deubiquitin inhibitor, which is a substance derived from lung cancer cells treated with radiation therapy. Microparticles cross the blood‐brain barrier and target and reprogram M2 macrophages at the same time, promoting effector T cell infiltration and the release of pro‐inflammatory cytokines, effectively inhibiting the development of brain metastases of lung cancer.

In this study, we observed that radiation‐treated cell‐released microparticles (RMPs) have greater effects to target and activate macrophages in vitro, compared to MPs derived from other cell culture conditions, including normal culture conditions, chemotherapy, and ultraviolet (UV) radiation. Moreover, we found that USP7 is highly expressed in both human and murine BRM, as well as in TAMs, indicating the potential of a USP7 inhibitor in reversing the brain ITME. To further improve the ability of RMPs to cross the BBB, we expressed an scavenger receptor‐B1 (SR‐B1)‐targeting peptide on the outer surface of RMPs through genetic engineering, as SR‐B1 receptor was reported to be expressed in BBB endothelial cells, M2Ф/microglial cells, and Lewis Lung Carcinoma (LLC) cells.^[^ ^23^ ^]^ Based on these principles, we constructed a genetically engineered and drug‐loaded P5091@RMPs‐R4F, which contains the RMP carrier, along with an SR‐B1‐targeting peptide, and a USP7 inhibitor. Compared with the original RMPs, the designed P5091@RMPs‐R4F infiltrated the BBB in much greater numbers, could specifically target F4/80^+^CD206^+^ M2Φ/microglial cells, and reduced the expression of CD206 in M2Φ in vitro. Following intravenous injection, P5091@RMPs‐R4F could effectively reprogram M2Φ by activating the MAPK signaling pathway through inhibition of USP7 enzyme activity, improve the ITME, and lengthen the survival rate of mice with BRM (**Figure** **1**). When combined with immune checkpoint blockade immunotherapy, the P5091@RMPs‐R4F significantly enhanced effector T‐cell infiltration and survival in mice. Therefore, this study demonstrates a new brain‐targeted delivery system and immune regulation targeting for the treatment of BRM.

## 2 Results

### 2.1 Plasmid Construction and Characterization of P5091@RMPs‐R4F

**Figure 2:**
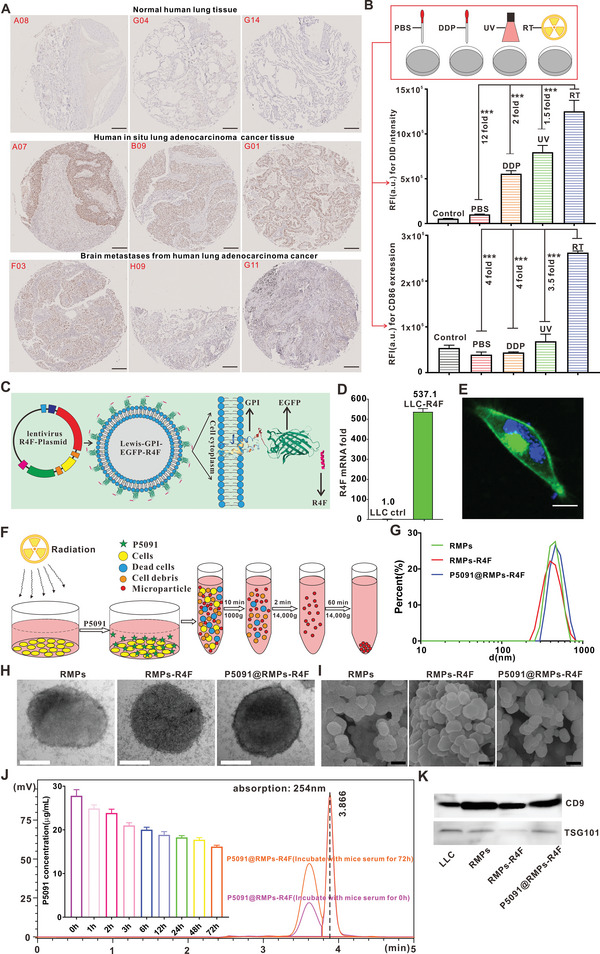
P5091@RMPs‐R4F construction and characterization. A) Immunohistochemistry was performed to investigate the expression of USP7 in human lung adenocarcinoma in situ and in brain metastases (the number in the image represents the location in the tissue chip). Scale bar: 200 µm. B) The vesicles obtained from different treatments of tumor cells were screened for their ability to target and reprogram macrophages. Statistical analysis was performed using unpaired *t*‐test (*n* = 3). The data are presented as the mean ± SEM (*n* = 3). *p < 0.05, **p < 0.01, ***p < 0.001. C) Schematic diagram of the strategy to anchor the peptides to the outer membrane of the cell. D) R4F‐GFP‐GPI mRNA expression in Lewis or Lewis‐R4F cells. E) Representative images for the location of R4F‐GFP‐GPI in cells by confocal imaging, scale bar: 20 µm. F) Schematic diagram for the procedure used to obtain the corresponding P5091@RMPs‐R4F. G) Size distribution of P5091@RMPs‐R4F by dynamic light scattering. H) Size analysis of P5091@RMPs‐R4F by transmission electron microscopy, scale bar: 200 nm. I) Representative SEM images of RMPs, RMPS‐R4F, P5091@RMPs‐R4F. Scale bar is 400 nm. J) HPLC to be used to identify the drug release characteristics of P5091@RMPs‐R4F. K) Expression of CD9 and TSG101 in RMPs, RMPs‐R4F, and P5091@RMPs‐R4F.

We previously reported that USP7 can regulate the ITME and reprogram M2Ф in subcutaneous tumor models of lung cancer in mice.^[^ ^22^ ^]^ However, the expression level of USP7 in BRM is unknown. Therefore, histological examination was performed for primary lung adenocarcinoma and associated brain metastases from patients. The results showed that USP7 was not only highly expressed in brain metastases of the commonly used murine Lewis tumor model (Figure S1, Supporting Information) but also in human primary lung adenocarcinoma (A07, B09, G01) and brain metastases (**Figure** **2**A, F03, H09, G11), indicating that USP7 is a potential target for the treatment of BRM. In order to effectively reprogram the ITME in the brain and deliver USP7 inhibitors, it is desirable to select a suitable drug delivery platform with a large loading capacity, good biocompatibility, and intrinsic immune‐regulating ability. For this purpose, we compared MPs produced by cells treated with DDP, UV, radiotherapy, and normal culture conditions in terms of MP production yield and their ability to target and reprogram M2Ф. The MP content produced by radiotherapy was similar to that of UV treatment and more than fivefold that of normal culture condition and DDP. To test their ability to target and reprogram M2Ф, the obtained MPs were labeled with DiD dye and incubated with BMDM‐derived M2Ф. Flow cytometry data showed that the fluorescence signal in the radiotherapy group was 1.5‐fold, 2‐fold, and 12‐fold the signals in the UV, DDP, and normal culture condition groups, respectively, suggesting that RMPs have the best M2Ф‐targeting capability among the tested groups. More importantly, the expression of MФ polarization marker CD86 in the radiotherapy group was 3.5‐fold, 4‐fold, and 4‐fold that of the UV, DDP, and normal culture condition groups, respectively, suggesting that RMPs have the strongest ability to polarize M2Ф among the tested groups. Taken together, we selected MPs produced by radiotherapy‐treated tumor cells for subsequent experiments, as they had the best effect to target and reprogram M2Ф, and produced a greater number of MPs (Figure 2B, Table S1, Supporting Information).

To improve the ability of RMPs to cross the BBB, we expressed the peptide R4F to mimic high‐density lipoprotein on the surface of LLC cells. To localize the R4F peptide to the surface of the cell membrane, a transferrin signal peptide sequence (MRLTVGALLACAALGLCLA) was added upstream of the peptide, and an EGFP sequence was added after R4F to allow tracing of the location of the R4F peptide. Meanwhile, a GPI membrane anchoring sequence (GGSSLQSTAGLLALSLSLLHLYC) was added at the end to anchor the R4F to the surface of the cell membrane. The overall plasmid design is shown in Figure 2C and the fusion gene sequence is shown in Table S2 (Supporting Information). After expressing the target fragment, confocal microscopy and RT‐PCR were used to determine the expression and localization of the EGFP fluorescence signal in LLC cells. It was found that EGFP was highly expressed in transfected LLC cells and mainly distributed around the cell membrane (Figure 2D,E). In order to verify the presence of R4F in the RMPs produced by transfected LLC after radiotherapy and to estimate the concentration of R4F‐EGFP‐GPI, we used GFP as an indicator of R4F‐EGFP‐GPI. Figure S2A (Supporting Information) shows that RMPs‐R4F displayed GFP signal, reflecting that R4F was enriched in RMPs. The concentration of R4F‐EGFP‐GPI was further quantified by calculating the grayscale values from Western blotting analysis with a standard curve made from GFP. As shown in Figure S2B (Supporting Information), R4F‐EGFP‐GPI accounted for ≈0.1% of the total RMPs‐R4F protein content. To enhance the ability of RMPs to polarize M2Ф and kill tumor cells, we also loaded RMPs with the USP7 inhibitor P5091; the acquisition strategy for P5091@RMPs‐R4F is shown in Figure 2F. Dynamic light scattering and transmission electron microscopy were used to identify the RMPs after radiotherapy (Figure 2G,H). The particle sizes of RMPs, RMPs‐R4F, and P5091@RMPs‐R4F were essentially the same, predominantly distributed between 400 and 600 nm, and these results were consistent with scanning electron microscopy analysis (Figure 2I). We calculated that P5091@RMPs‐R4F contained nearly 0.1 mg of P5091 per 1 mg of protein content using HPLC (Figure S2C, Supporting Information).

Next, the serum stability of P5091@RMPs‐R4F was examined using dual dye‐labeled RMPs, in which CFSE was mainly used to label the contents of RMPs. The rationale is that CFSE can label proteins in living cells, thus RMPs released from CFSE‐labelled cells would contain a large number of CFSE‐labelled proteins. DIR dye was selected to label the outer surface of RMPs. When the structure of the RMPs is destroyed, the contents will leak, resulting in the loss of fluorescence co‐localization of the contents and the outer surface of RMPs. For this experiment, we incubated RMPs with mouse serum and fetal bovine serum for different periods, and then used native SDS‐PAGE to verify the serum stability of RMPs. The results showed that the fluorescence of CFSE and DIR was highly co‐localized throughout the tested incubation time periods (Figure S3, Supporting Information), suggesting that RMPs remain stable in structure. We further investigated the release of P5091 loaded in RMPs‐R4F in the presence of mouse serum using HPLC. A sustained release profile was observed (Figure 2J), and after 72 h incubation, the concentration of P5091 was about two‐thirds of the original concentration, showing that RMPs‐R4F can release hydrophobic drugs such as P5091. Additionally, the classical molecular markers of MPs such as CD9 and TSG101 were also expressed in RMPs, RMPs‐R4F, and P5091@RMPs‐R4F (Figure 2K).

In addition, CCK‐8 assays confirmed that RMPs‐R4F@ P5091 had a stronger cytotoxic effect on LLC cells than RMPs‐R4F (Figure S4, Supporting Information). To further demonstrate the high specific targeting ability of RMPs‐R4F to SR‐B1, we selected the SR‐B1‐negative cell line IDIA7 and positive cell line mSR‐B1 to confirm the expression of R4F on the surface of RMPs. First, the membranes of RMPs‐R4F were stained with PKH67 green fluorescent dye, and then incubated with IDIA7 and mSR‐B1 for 3 h. Confocal imaging and flow cytometry results showed that the fluorescence intensity of the mSR‐B1 cells was 2.5‐fold that of the IDIA7 cells, which confirmed the specific targeting of RMPs‐R4F for the SR‐B1 receptor (Figure S5, Supporting Information). The above results confirmed that R4F was mainly expressed on the outer membrane surface of RMPs and had a strong loading capacity for P5091, which could effectively target cells expressing SR‐B1 receptor.

### 2.2 Tumor/TAM Targeting and Reprogramming Ability of P5091@RMPs‐R4F

Brain macrophages and microglia play an important role in BRM. It has been reported that the SR‐B1 receptor is highly expressed on the surface of phagocytes and some type of tumors,^[^ ^22^, ^25^ ^]^ so we determined whether RMPs‐R4F could target macrophages/microglia and LLC cells in vitro. To achieve this, we used the BV2 microglial cell line, M2Φ and M1Φ differentiated from mouse bone marrow cells, and LLC cells to investigate cell type‐specific targeting. Figure S6 (Supporting Information) shows that compared with M1Φ and M2Φ, BV2 cells expressed higher levels of the SR‐B1 receptor.

**Figure 3:**
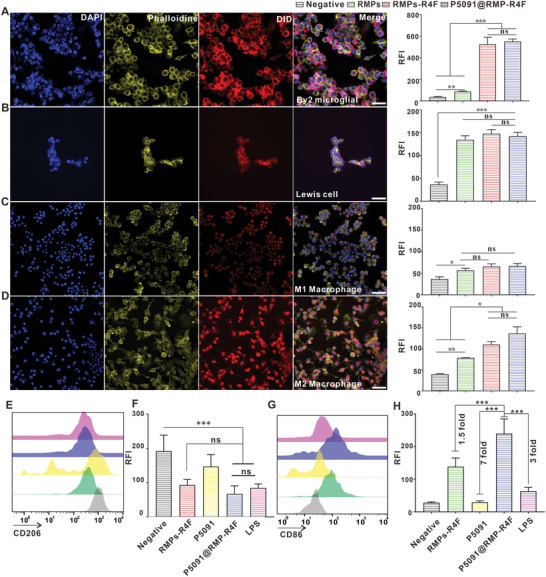
P5091@RMPs‐R4F targeting and reprograming M2Φ. A–D) Representative images of RMPs‐R4F uptake in BV2 microglia, LLC cells, M1Φ, and M2Φ, and the relative fluorescence intensities of the above‐mentioned cells after incubation with different DiD‐stained microvesicles by flow cytometry. Scale bar: 50 µm. E,G) Representative expression of CD206 and CD86 in M2Φ after incubation with different RMPs. F,H) Relative fluorescence intensity of CD206 and CD86 in M2Φ after incubation with different RMPs by flow cytometry. Statistical analysis was performed using unpaired *t*‐test (*n* = 3). The data are presented as the mean ± SEM. *p < 0.05, **p < 0.01,***p < 0.001, and ns: not significant.

To identify whether RMPs‐R4F could specifically target M2Φ, we incubated the previous mentioned cells with DiD‐stained RMPs, RMPs‐R4F, or P5091@RMPs‐R4F. Compared with M1Φ and LLC cells, confocal imaging in **Figure** **3**A–D shows that M2Φ and BV2 cells took up much more RMPs‐R4F. Flow cytometry results showed that M1Φ took up equal number of RMPs, RMPs‐R4F, and P5091@RMPs‐R4F (Figure 3C). However, R4F peptide enhanced RMP uptake in M2Φ by more than 1.5 times (Figure 3D), in BV2 microglia by more than 3 times (Figure 3A), and in tumor cells by more than 2 times (Figure 3B). The packaging of P5091 did not affect the targeting properties of P5091@RMPs‐R4F compared with RMPs‐R4F.

To determine whether the loading of P5091 in P5091@RMPs‐R4F could enhance the M2Φ polarization ability of RMPs, we used CD206 and CD86 as representative markers of the polarization of MΦ to identify the reprogramming ability of the tested groups. The expression level of CD206 in the LPS‐positive control group, RMPs‐R4F group, and single drug P5091 group was 1.2 fold, 1.3 fold, and 2 fold that of the P5091@RMPs‐R4F group (Figure 3E,F). In addition, P5091@RMPs‐R4F can effectively promote the expression of CD86 in M2Φ, achieving levels that were 1.5 fold that of the RMPs‐R4F group, 7 fold that of the P5091 group, and 3 fold that of the LPS group (Figure 3G,H). These experimental results revealed that R4F could promote the uptake of RMPs by M2Φ, BV2 microglia, and LLC cells. Meanwhile, P5091 loaded in RMPs‐R4F did not affect its targeting function and could reprogram M2Φs more effectively compared with RMPs‐R4F and P5091 alone.

### 2.3 Crossing the Mimetic BBB by P5091@RMPs‐R4R In Vitro and In Vivo

To shed light on whether RMPs‐R4F and P5091@RMPs‐R4R cross the BBB, we constructed an in vitro mimetic BBB model using SR‐B1‐expressing bEnd.3 endothelial cells.^[^ ^26^ ^]^ BEnd.3 endothelial cells were incubated in the upper chambers of Transwell inserts, while M2Φ/microglia and LLC cells were simultaneously incubated in the lower chambers to take up RMPs‐R4F that cross the mimetic BBB (Figure S7A, Supporting Information). PKH67‐stained RMPs, RMPs‐R4F, or P5091@RMPs‐R4F were incubated in the upper chamber. After 24 h, the lower chamber cells were separated for flow cytometry identification. The fluorescence intensity in M2Φ/microglia (BV2) and LLC cells incubated with P5091@RMPs‐R4F were similar compared to those incubated with RMPs‐R4F and more than 3 times higher compared to those incubated with RMPs. Therefore, RMPs‐R4F could effectively cross the BBB and P5091 loading did not affect their ability to cross the BBB (Figure S7B–E, Supporting Information). To determine whether RMPs‐R4F crossed the BBB by transendocytosis, we incubated RMPs or RMPs‐R4F (both stained with DiO dye) with bEnd.3 cells for 6 h and labeled the lysosomes with lysosomal dye. Confocal imaging results (Figure S7F,G, Supporting Information) showed that both RMPs and RMPs‐R4F existed in the cytoplasm of bEnd.3 cells, likely in the form of complete RMPs. However, they did not colocalize with lysosomes, thus demonstrating the role of RMPs in crossing the BBB. Additionally, we identified that SR‐B1 was also highly expressed in endothelial cells at the BBB in mice, which implies that RMPs‐R4F can undergo transendocytosis in vivo (Figure S7H, Supporting Information).

**Figure 4:**
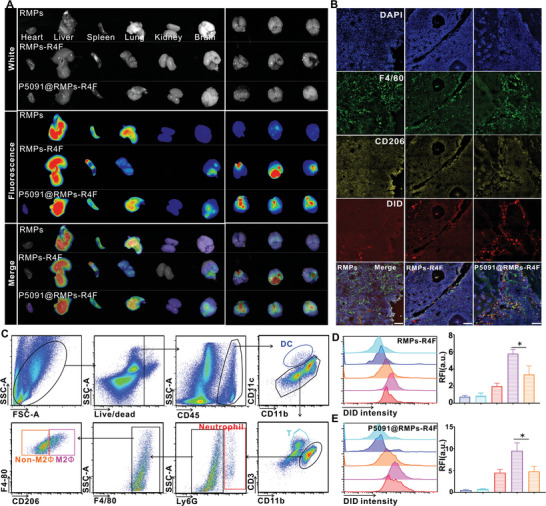
P5091@RMPs‐R4F cross the blood brain barrier in vivo. A) A whole body imaging system was used to analyze the distribution of DiD dye‐stained RMPs, RMPs‐R4F, or P5091@RMPs‐R4F in different organs 24 h after *i.v*. injection. B) Immunofluorescence was performed to detect the CD206‐positive macrophages as an indication of the targeting ability of RMPs, RMPs‐R4F, and P5091@RMPs‐R4F injected into the tail vein. Images were obtained by confocal microscopy. Red‐DiD, Yellow‐CD206, Green‐F4/80, Blue‐DAPI. Scale bar: 100 µm. Data are presented as the mean ± SEM (*n* = 3). C) Gating strategy to distinguish different immune cell types. D,E) Fluorescence intensity of different immune cell types by flow cytometry. Statistical analysis was performed using one‐way ANOVA with Tukey's multiple comparison test (*n* = 3). Data are presented as the mean ± SEM. *p < 0.05, **p < 0.01, ***p < 0.001.

To characterize the crossing of the BBB by RMPs‐R4F in vivo, we first constructed a BRM model in mice. We stained RMPs, RMPs‐R4F, and P5091@RMPs‐R4F with DiD dye, and then injected C57 mice in the tail vein. After 24 h, all organs of the mice were taken out for *ex vivo* imaging analysis. The results showed that P5091@RMPs‐R4F were distributed in all organs and were especially enriched in the liver, spleen, lung, and brain (**Figure** **4**A). Ex vivo images further confirmed that RMPs‐R4F and P5091@RMPs‐R4F could more efficiently aggregate in the brain than in other organs (Figure 4A). To verify that RMPs‐R4F and P5091@RMPs‐R4F target mouse brain M2Φ after crossing the BBB, antibodies against F4/80 and CD206 were used to label mouse M2Φ/microglia. Immunofluorescence analysis of brain tissues sections showed that the signals carried by the RMPs‐R4F or P5091@RMPs‐R4F could co‐localize with F4/80^+^CD206^+^ M2Φ, demonstrating that RMPs‐R4F can effectively cross BBB and target tumor‐infiltrating M2Φ/microglia (Figure 4B).

To further quantify the phagocytic ability of infiltrating immune cells in the brain, we calculated the fluorescence intensity of different infiltrating immune cells in the brain by flow cytometry. The gating strategy is shown in Figure 3C. Compared with non‐M2 cells, M2Φ and neutrophils, we found that F4/80^+^CD206^+^ M2Φ had the strongest intensity after *i.v*. injection of RMPs‐R4F and P5091@RMPs‐R4F (Figure 4D,E), while RMPs showed no differences in different immune cells (Figure S8, Supporting Information), thus confirming the targeting ability of RMPs‐R4F and P5091@RMPs‐R4F.

### 2.4 Therapeutic Effect of P5091@RMPs‐R4F in a BRM Model

**Figure 5:**
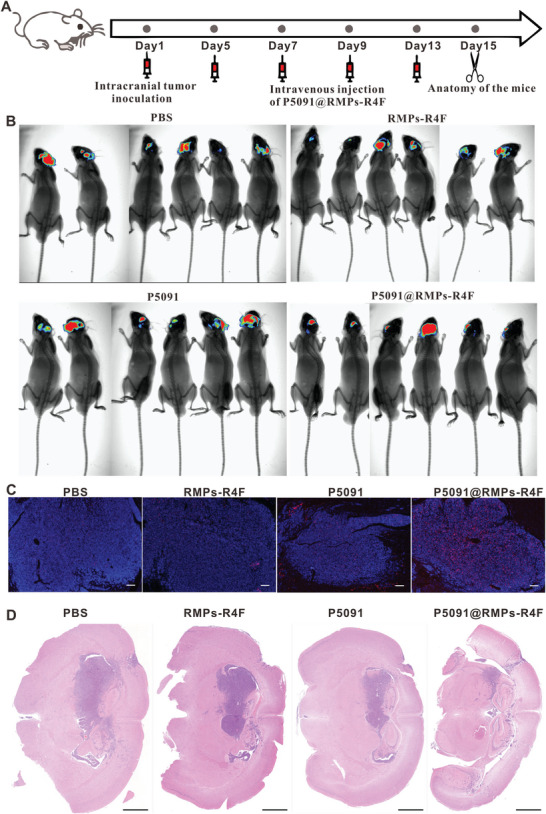
Therapeutic effect of P5091@RMPs‐R4F in a lung cancer brain metastasis model. A) Schematic diagram of model establishment time and drug injection time. B) Whole body images showing the growth of luciferase‐expressing LLC cells in the brain after various drug treatments. C) Representative images of TUNEL staining to observe tumor cell death after the indicated treatments. Scale bar: 100 µm. D) Representative images of HE staining to observe the tumor area in the brain after the indicated treatments. Scale bar: 1 mm.

To verify the in vivo therapeutic effect of P5091@RMPs‐R4F, we used LUC cells to construct the BRM model. Since RMPs that do not express R4F do not localize in the central nervous system, we did not use RMPs to treat mice. On days 5, 7, 9, and 13, RMPs‐R4F, P5091@RMPs‐R4F were given through the tail vein (**Figure** **5**A), while equal doses of P5091 were simultaneous administrated through intraperitoneal injection as s control group. On the second day after treatment, the growth of lung cancer cells in the brains of mice was observed by intraperitoneal injection of luciferase substrate. Compared with the other treatments, the P5091@RMPs‐R4F group had the slowest growth of BRM (Figure 5B). The luminescence intensity in the brains of mice in the P5091@RMPs‐R4F treatment group was significantly lower compared to other groups (Figure S9, Supporting Information). To further confirm the therapeutic effect of P5091@RMPs‐R4F, we used LLC cells to construct BRM. After the same treatment, TUNEL staining and HE staining were used to observe the growth of lung cancer in the brain. TUNEL staining showed that P5091@RMPs‐R4F could promote the apoptosis of most tumor cells in the tumor region (Figure 5C), and HE staining showed that P5091@RMPs‐R4F delayed the growth of lung cancer cells in the brain to the greatest extent (Figure 5D). In conclusion, compared with P5091 and RMPs‐R4F alone, P5091@RMPs‐R4F demonstrates an effective therapeutic effect on BRM.

**Figure 6:**
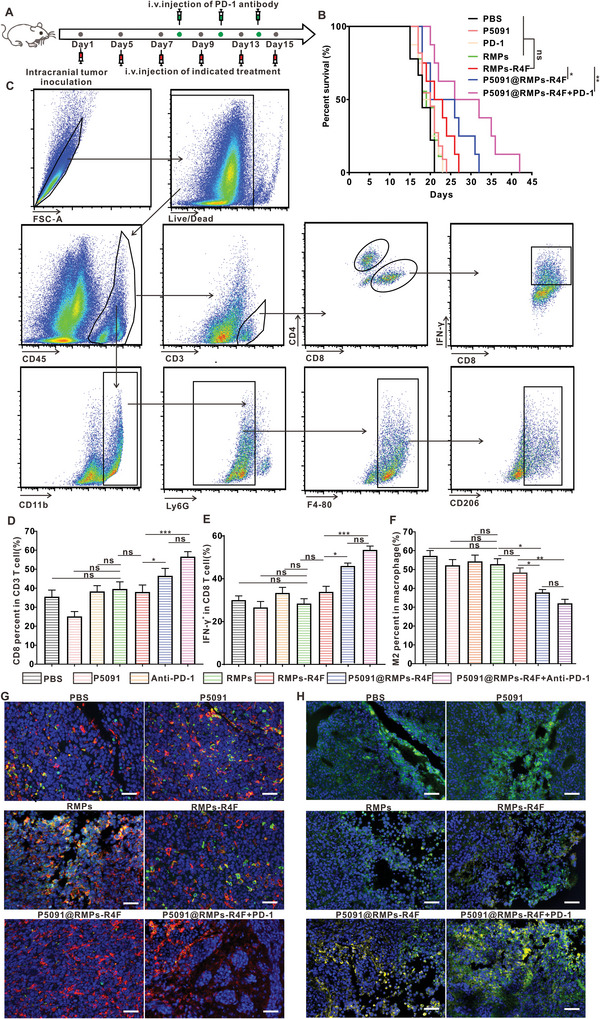
Immune cell invasion in the tumor environment after different treatments. A) Schematic diagram of model establishment time and drug injection time. B) Survival statistics for different treatment groups (*n* = 10 mice). C) Gating strategy for flow cytometry of immune cells and representative results from the indicated treatment. D–F) CD8^+^ T cell percentages among CD3^+^ cells, CD8^+^ IFN‐*γ* ^+^ T cell percentages among CD8^+^ T cells, and M2Φ percentages among F4/80^+^ macrophages from the indicated treatments. Data are presented as the mean ± SEM (*n* = 6 mice). G) Representative immunofluorescence images from the indicated treatments. Blue‐DAPI, Red‐F4/80, Green‐CD206. Scale bar: 50 µm. H) Representative immunofluorescence images from the indicated treatments. Blue‐DAPI, Green‐CD8, Yellow‐IFN‐*γ*. Scale bar: 50 µm. Statistical analysis was performed using log‐rank Mantel–Cox test for (B), and one‐way ANOVA with Tukey's multiple comparison test for (D, E, F). Data are presented as the mean ± SEM. *p < 0.05, **p < 0.01, ***p < 0.001, and ns: not significant.

As PD‐1 antibody therapy has shown potential to treat lung metastases, and we found that P5091 treatment could enhance PD‐L1 expression,^[^ ^22^ ^]^ we hypothesized that P5091@RMPs‐R4F combined with PD‐1 antibody therapy could achieve greater efficacy. To investigate the therapeutic effect of P5091@RMPs‐R4F combined with PD‐1 antibody treatment on inhibiting BRM, we conducted statistical analysis on the survival rate of mice after the indicated treatments (**Figure** **6**A). Remarkably, P5091@RMPs‐R4F combined with PD‐1 antibody could significantly prolong the survival rate of mice compared with P5091@RMPs‐R4F and the other groups (Figure 6B).

### 2.5 Mechanism of the Combination Treatment of P5091@RMPs‐R4F and PD‐1 Antibody in BRM

Our previous experiments showed that P5091@RMPs‐R4F could reprogram M2Φ and cross the BBB to specifically target M2Φ. Based on this, we speculated that P5091@RMPs‐R4F combined with PD‐1 antibody could reverse the ITME and enhance the infiltration of effector CD8^+^ T cells to target BRM. To test this hypothesis, immune cells were isolated from the brains of mice after different treatments, and the infiltration of immune cells in different treatment groups was analyzed by flow cytometry (Figure 6C). The results showed that P5091@RMPs‐R4F alone could promote the infiltration of effector T cells, which were present at 1.5 times the number in the control group and RMPs‐R4F groups, and twice that of the P5091 group (Figure 6D). The proportion of IFN‐*γ* positive T‐cells in the P5091@RMPs‐R4F group was significantly higher compared to the other treatment groups (Figure 6E). The promotion of effector T‐cell infiltration by P5091@RMPs‐R4F was further verified by immunofluorescence analysis, as shown in Figure 6H. When combined with PD‐1 antibody, the infiltration of effector T‐cells was even higher, demonstrating that P5091@RMPs‐R4F can promote the immune response mediated by PD‐1 antibody. In addition, the proportion of M2Φ in mice treated with P5091@RMPs‐R4F with or without PD‐1 antibody was about 40–60% of those in the PBS, PD‐1 antibody, and P5091 groups. There was no significant difference in the proportion of M2Φ in mice given P5091@RMPs‐R4F compared to mice given P5091@RMPs‐R4F combined with PD‐1 antibody (Figure 6F). However, the M2Φ proportion in either of the two groups was lower than that in the RMPs‐R4F group, indirectly showing that P5091@RMPs‐R4F had a certain ability to reprogram M2Φ (Figure 6F). To further confirm that P5091@RMPs‐R4F could reprogram M2Φ, we performed immunofluorescence on BRM slices from different treatment groups. CD206 and F4/80 staining showed that the proportion of M2Φ infiltration was the lowest in the mice given P5091@RMPs‐R4F with or without PD‐1 antibody treatment (Figure 6G). The above experimental results confirmed that P5091@RMPs‐R4F can improve the ITME by reprogramming M2Φ, thus promoting the infiltration of effector T‐cells and exerting anti‐tumor immune effects. Furthermore, when combined with PD‐1 antibody, P5091@RMPs‐R4F had an even stronger ability to reprogram the ITME.

**Figure 7:**
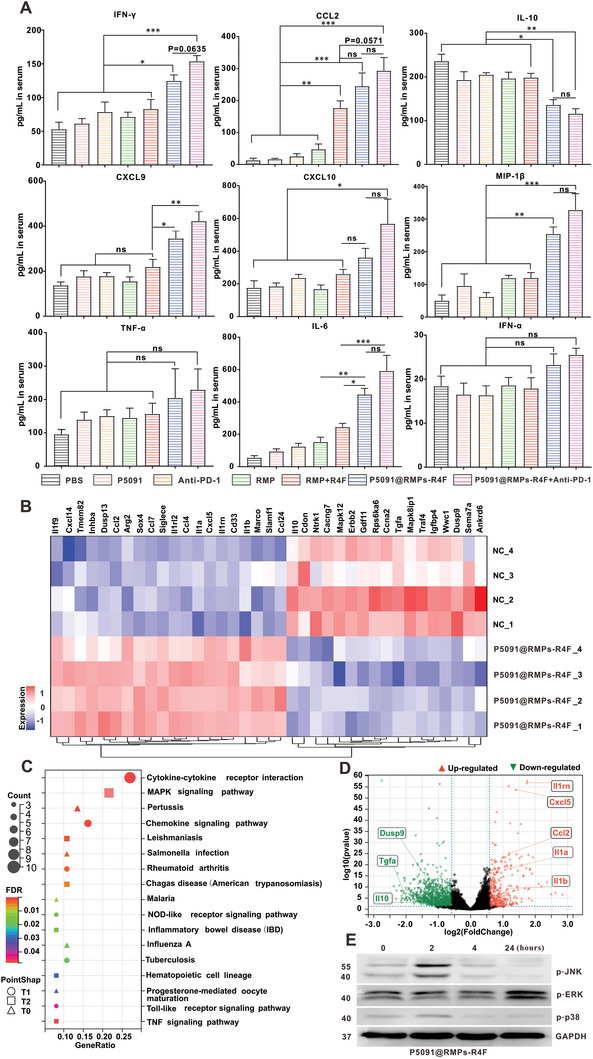
Cytokine detection after treatment and molecular mechanisms of macrophage reprogramming induced by P5091@RMPs‐R4F. A) Cytokine concentrations in serum from the indicated treatment groups as measured by flow cytometry (*n* = 6 mice). B) Heat map illustrating the differentially expressed M1‐and M2‐related genes in TAMs in the P5091@RMPs‐R4F group and the Control group based on RNA sequencing results. C) KEGG analysis identifying the 17 most enriched pathways based on the differentially expressed genes of the two groups. D) Volcano plots of the differentially expressed genes. Red dots show significantly up‐regulated genes, and green dots show significantly down‐regulated genes. E) Western blotting of p‐JNK, p‐ERK, p‐p38, and GAPDH in IL‐4/13‐BMDM M2 cells treated with P5091@RMPs‐R4F at the indicated time points. Statistical analysis was performed using one‐way ANOVA with Tukey's multiple comparison test for (A). Data are presented as the mean ± SEM. *p < 0.05, **p < 0.01, ***p < 0.001, and ns: not significant.

To further explore the mechanism of P5091@RMPs‐R4F in systemic anti‐tumor immunotherapy, we measured the serum levels of cytokines associated with anti‐tumor immunity, as shown in **Figure** **7**A. Compared with other groups, P5091@RMPs‐R4F with or without PD‐1 antibody showed higher levels of IFN‐*γ*, which is consistent with high infiltration of IFN‐*γ* expressing CD8^+^ T cells, and PD‐1 antibody enhanced the effect of P5091@RMPs‐R4F on IFN‐*γ* secretion. Furthermore, the secretion of IL‐10 by M2Φ was significantly decreased and the T cell chemokines CXCL9/CXCL10 were significantly increased in the P5091@RMPs‐R4F groups with or without PD‐1 antibody. At the same time, serum levels of proinflammatory cytokines such as CCL2, IL‐6, and MIP‐1*β* were also significantly elevated, indicating an enhancement of the systemic anti‐tumor immune response after P5091@RMPs‐R4F treatment.

### 2.6 Mechanism of P5091@RMPs‐R4F in Reprogramming TAMs

To investigate the molecular mechanisms of the P5091@RMPs‐R4F‐induced in vivo reprogramming of M2Φ, TAMs from BRM treated with P5091@RMPs‐R4F and controls were sorted by flow cytometry, and their mRNA changes were analyzed by subsequent RNA sequencing (RNA‐seq). As shown in the heat map of Figure 7B and the volcano map of Figure 7D, the anti‐inflammatory cytokine (IL‐10) related to M2Φ were highly down‐regulated, while the pro‐inflammatory cytokines related to M1 macrophages (IL‐1*α*/*β*) were up‐regulated in P5091@RMPs‐R4F‐treated samples compared to controls, which was consistent with the results of Figure 7A. These data further confirm that P5091@RMPs‐R4F can reprogram macrophages to release more pro‐inflammatory cytokines and reverse the ITME of the brain. To further evaluate the effect of P5091 loading in P5091@RMPs‐R4F on TAM reprogramming, we also used the Kyoto Encyclopedia of Genes and Genomes (KEGG) to identify the enriched canonical signaling pathways in TAMs. As shown in Figure 7C, the M1‐related MAPK signaling pathway was significantly enriched in the P5091@RMPs‐R4F‐treated group compared to the control group, which is consistent with previous research.^[^ ^22^ ^]^ Western blotting in Figure 7E confirmed that the phosphorylation levels of MAPK pathway proteins (JNK, ERK, and p38) were increased in P5091@RMPs‐R4F‐treated M2Φ induced from BMDMs. Collectively, P5091@RMPs‐R4F retained its ability to specifically inhibit the USP7 activated p38 MAPK pathway, resulting in TAM reprogramming.

## 3 Discussion

Previously, it has been shown that radiotherapy could be combined with neuron‐derived vesicles modified with RGD peptides containing PD‐L1‐siRNA for efficacy in the treatment of brain tumors in animal models.^[^ ^27^ ^]^ However, radiotherapy has potential to damage the normal brain tissue adjacent to the cancer. We previously reported that the RMPs produced by tumor cells can exhibit a bystander effect to promote the death of adjacent tumor cells, mainly through ferroptosis,^[^ ^15^ ^]^ while the supernatant produced by radiotherapy‐treated tumor cells can also effectively reverse the ITME.^[^ ^28^ ^]^ According, we put forward the concept of indirect radiotherapy, which provides hope for patients who are unable to receive traditional radiotherapy, such as advanced pleural effusion, ascites, and multiple brain metastases.

MPs produced by host normal cells or tumor cells regulate multiple physiological and pathophysiological processes, such as tissue repair and promotion of tumor tissue proliferation.^[^ ^29^ ^]^ The RMPs produced by some types of tumors after radiotherapy have been reported to promote the invasive characteristics of the adjacent non‐irradiated tumor cells,^[^ ^30^ ^]^ but not all the RMPs produced after radiotherapy will cause this phenomenon. Kang et al. found that when cells were treated with radiotherapy and chemotherapy, RAB22A protein can inactivate RAB7 and other proteins, promote the formation of ER‐derived a typical autophagosomes encapsulated with activated STING protein and the secretion of external vesicles, thus promoting anti‐tumor immunity.^[^ ^31^ ^]^ Our previous study found that radiotherapy vesicles derived from LLC cells can induce ferroptosis of tumor cells, promote macrophage reprogramming and neutrophil activation, but do not cause proliferation of adjacent tumor cells.^[^ ^15^, ^32^ ^]^ Thus, RMPs produced from radiotherapy can be used as a drug carrier for targeting ITME.

MPs produced by irradiated tumor cells carry tumor antigens and immunogenic proteins, such as Heat shock proteins (HSP) family proteins, which can be used as prophylactic and therapeutic vaccines through immunization to treat established tumors.^[^ ^33^, ^34^ ^]^ Baharom et al. reported that a self‐forming nanovaccine containing adjuvant and antigen was more effective against tumors when administered through the tail vein than subcutaneous immunization, as the intravenous vaccine can reach both the spleen and lymph nodes, activating more secondary lymphatic organs and promote tumor regression via antigen‐specific CD8^+^ T cells and type I interferon‐dependent modulation of the TIME.^[^ ^35^ ^]^ In this study, the use of *i.v*. injection of drug‐carrying radiotherapy MPs in the treatment of lung cancer BRM could achieve vaccine efficacy to some extent by carrying tumor antigens to the spleen (Figure 4A). In addition, radiotherapy vesicles injected by tail vein can also directly act on the local tumor to kill, or can play a role of killing two birds with one stone.

Related studies have reported that radiotherapy can stimulate the expression of PD‐L1 in tumor cells and a large number of RMPs released by tumor cells also contain this molecule, which may dampen the killing effect of cytotoxic T cells.^[^ ^36^ ^]^ For this reason, we combined *i.v*. injection of P5091@RMPs‐R4F with *i.p*. injection of PD‐1 antibody by shielding PD‐L1/PD‐1 signal axis in peripheral or lymph nodes, thus further promoting the anti‐tumor immunity of RMPs. It has been reported that PD‐1 antibody alone or combined with other therapies can improve the survival rate of lung cancer patients with brain metastasis.^[^ ^37^ ^]^ Additionally, PD‐1 antibody is mainly applied to activate cytotoxic T cells expressing PD‐1 molecules, and PD‐1 antibody can partially bind to PD‐1‐expressing CD8^+^ T cells before the cell infiltrating the brain. Therefore, PD‐1 antibody can play a certain role against brain tumors while not infiltrating the brain. Recent studies by Ye at al also reported that PD‐1 antibody could act on tumor‐memory TCF‐1^+^PD‐1^low^ T cells in tumor draining lymph nodes, promoting the proliferation and tumor invasion of these cells, instead of acting on the tumor in situ.^[^ ^38^ ^]^ The combination of PD‐1 antibody and P5091@RMPs‐R4F can effectively enhance the anti‐tumor immune effect and significantly prolong the survival time of mice affected by BRM. Therefore, PD‐1 antibody combined with P5091@RMPs‐R4F provides a potential novel strategy for future clinical immunotherapy against BRM in patients.

Although USP7 inhibitors combined with RMPs can effectively kill tumor cells in vitro, the number of RMPs produced by this system that can reach the CNS is limited. Therefore, in this study, we did not investigate the direct killing effect of the vesicle system loaded with USP7 inhibitors on BRM. Instead, by using P5091@RMPs‐R4F, we found that the reprogramming of M2Φ could effectively reverse the ITME of BRM, thereby inhibiting tumor development. The results presented herein demonstrate that the RMPs possess a certain ability to reprogram M2Φ, an effect that is further consolidated by combination with USP7 inhibition. Taken together, these results demonstrate the advantages of RMPs vectors combined with USP7 inhibitors.

To address the issue of crossing of the BBB, in addition to traditional methods,^[^ ^39^ ^]^ newly reported strategies include cholesterol‐modified drugs,^[^ ^40^ ^]^ human serum albumin,^[^ ^41^ ^]^ and single‐stranded DNA nanotubes,^[^ ^42^ ^]^ providing a variety of options for brain delivery of therapeutic drugs. Here, we chose to modify RMPs with the R4F peptide as the targeting ligand. Ruhland et al. have found that MPs released by tumor cells could be endocytozed in a form of complete vesicle into migratory DCs, which then transferred the MPs to CD8*α* ^+^ DCs in draining lymph nodes, proving that MPs could undergo the transendocytosis process in a complete form in some cells.^[^ ^43^ ^]^ In addition, SR‐B1 has been reported to be a strategy for transendocytosis of BBB to transport LDL to brain macrophages, which requires the assistance of DOCK4 protein expressed by the endothelial cells themselves.^[^ ^44^ ^]^ Therefore, the binding of MPs to SR‐B1 ligand R4F can promote the stronger ability of MPs to cross the BBB. Besides, this approach has several putative advantages: 1) the R4F peptide can simultaneously target tumor cells and M2Φ with high SR‐B1 receptor expression, thus killing two birds with one stone. 2) The R4F peptide can target the SR‐B1 receptor on endothelial cells of the BBB and promote RMP accumulation in the CNS by transendocytosis (Figure S7, Supporting Information). 3) By genetic engineering, a large number of R4F peptides can be displayed on the surface of RMPs to achieve a multivalent targeting effect. Compared with other BBB‐crossing strategy, the use of R4F is a simple process with good biocompatibility and represents the ideal approach to promoting clinical translation.

Although our results show that the MP content produced by radiotherapy has advantages compared with that of normal condition and chemotherapy, additional approaches are still required to assist the generation of RMPs for clinical translation. One option is the use of cell nanoperforation, a means to promote the clinical transformation of external MPs, which has been reported to scale‐up the production of small MPs by more than 50 fold.^[^ ^45^ ^]^ The strategy of combining cell nanoperforation will be considered in our future studies to enhance the output of RMPs.

In conclusion, P5091@RMPs‐R4F can effectively cross the BBB, target TAMs and LLC cells, promote the mass death of tumor cells at the site of BRM, effectively improve the ITME, and enhance the application of macrophage polarization drugs in the treatment of BRM.

## 4 Experimental Section

### Mice

C57BL/6 female mice were obtained from the Hunan Slyke Jingda Laboratory Animal Co. LTD (Hunan, China, Experimental animal quality certificate No. 430727210103000762). CX3CR1^GFP/+^transgenic mice were purchased from Jackson laboratories (Strain#: 005582). All the mice were bred and maintained in a specific pathogen‐free (SPF) barrier facility. All animal studies were approved by the Hubei Provincial Animal Care and Use Committee and followed the experimental guidelines of the Animal Experimentation Ethics Committee of the Huazhong University of Science and Technology.

### Cells

Mouse bEnd.3, LLC, and BV2 cells were obtained from the China Center for Type Culture Collection (Wuhan, China). The luciferase‐Lewis tumor cell line, RFP‐LLC cells, and R4F‐GFP‐LLC cells were established in the lab. All cell lines were treated with 25 µg mL^−1^ Plasmocin (InvivoGene, Toulouse, France) for at least two weeks and were mycoplasma‐negative as determined by MycoProbe Mycoplasma Detection Kit (R&D Systems, Minneapolis, MN, USA). Cells were grown in Dulbecco's Modified Eagle's Medium (DMEM) (Gibco, Grand Island, NY, USA) containing 10% Fetal Bovine Serum (FBS) (Gibco, Grand Island, NY, USA) and 1% penicillin/streptomycin solution. The ldlA7 and ldlA (mSR‐B1) cell lines were gifts from Dr. Monty Krieger (Massachusetts Institute of Technology, Cambridge, MA). The cell culture medium for ldlA7 cells was Ham's F‐12 media with 2 mm L‐glutamine, 100 U mL^−1^ penicillin‐streptomycin, and 5% FBS. For the culture of ldlA (mSR‐B1) cells, similar conditions were used, except that 300 µg mL^−1^ geneticin was also added.

### Lentivirus Construction and LLC Cell Transfection

The DNA sequences for R4F‐GFP‐GPI expression are listed in the Supporting Information, mainly comprising the sequences for the signal peptide (MRLTVGALLACAALGLCLA), one R4F peptide, one EGFP, and a GPI anchor. Lentivirus containing a purinomycin resistance gene was constructed by Shanghai Jikai Biotechnology Co., LTD. The virus titer used to transfect LLC cells was 1 × 10^7^.

### Immunohistochemistry of Human Lung Cancer Tissue

Tissue microarrays were constructed by Shanghai Wellbio Biotechnology Co., Ltd (Wellbio Biotechnology Co., Shanghai, China). Patient samples from test and validation cohorts were paraffin‐embedded, sectioned, and stained with hematoxylin‐eosin (HE) by pathologists to confirm the diagnoses. Fixed points that displayed the most typical histological characteristics were marked under a microscope. Cores with diameters of 1.0 mm from per‐donor blocks were transferred into a recipient block microarrayer, and each dot array contained fewer than 220 dots. Four micrometer‐thick sections were cut from the recipient block and transferred to glass slides using an adhesive tape transfer system for UV cross linkage.

### Preparation of Microparticles (MPs) Processed in Different Ways

A conventional BCA protein quantitation assay was used to measure protein concentration. To prepare Cisplatin (DDP)‐MPs, 6 × 10^6^ mL LLC cells were cultured with 40 µmol L^−1^ DDP (Sigma, USA) for 72 h. The medium was collected, centrifuged, and processed by ultrafiltration. The resultant protein concentration was 0.26 mg mL^−1^. To prepare RMPs, 6 × 10^6^ mL LLC cells were irradiated with a single dose of 20 Gy by 6‐MV X‐rays (600 MU min^−1^, Trilogy System Linear Accelerator, Varian Medical Systems). After incubation for 72 h, the medium was collected, centrifuged, and processed by ultrafiltration, with a protein concentration of 1.12 mg mL^−1^. To prepare UV‐MPs, 6 × 10^6^ mL LLC cells were exposed to UV (UVB, 300 Jm^−2^) irradiation for 20 min. After 72 h of incubation, supernatants were collected, centrifuged, and processed by ultrafiltration, with a protein concentration of 1.13 mg mL^−1^.

### RT‐PCR of LLC and LLC‐R4F Cells

RNA extraction of LLC cells was performed using Trizol reagent according to the manufacturer's protocol. Reverse transcription was performed using the HiScript III RT SuperMix for qPCR (+gDNA wiper) from Vazyme, according to the manufacturer's protocol. The forward primer was CGCTAAGTTCTGGGACGGTG and the reverse primer was CTCGATGTTGTGGCGGATCT.

### Isolation of RMPs, RMPs‐R4F, and P5091@RMPs‐R4F

A total of 5 × 10^6^ cells were plated into 10 cm cell culture dishes and irradiated with a single dose of 20 Gy by 6‐MV X‐rays (600 MU min^−1^, Trilogy System Linear Accelerator, Varian Medical Systems). The medium was then replaced with 20 mL of complete medium (DMEM or RPMI 1640 with or without P5091 (purchased form Selleck), based on the needs of each cell line). After 72 h, the medium was collected and centrifuged at 1000 *g* for 10 min and then 14 000 g for 2 min to remove tumor cells and debris. Then, the supernatant was centrifuged at 14 000 *g* for 1 h at 4 °C to isolate RMPs, RMPs‐R4F, or P5091@RMPs‐R4F. The pellet (containing RMPs, RMPs‐R4F, or P5091@RMPs‐R4F) was washed twice with sterile 1 × PBS and resuspended in sterile 1 × PBS for animal experiments or resuspended in complete medium for cell experiments.

### Quantification of RMPs, RMPs‐R4F, and P5091@RMPs‐R4F

The protein concentrations of RMPs, RMPs‐R4F, and P5091@RMPs‐R4F were measured. After washing, RMPs, RMPs‐R4F, and P5091@RMPs‐R4F were lysed with radioimmunoprecipitation assay (RIPA) buffer at 4 °C for 30 min and then centrifuged for 30 min at 12 000 *g* at 4 °C. The supernatant containing the total protein was transferred to a new centrifuge tube. Protein was quantified using the BCA Protein Assay Kit (Thermo Fisher Scientific) in accordance with the manufacturer's protocol.

### Transmission Electron Microscopy (TEM) and Scanning Electron Microscopy (SEM)

P5091@RMPs‐R4F were observed by TEM. P5091@RMPs‐R4F in suspension were stained with 2% phosphotungstic acid solution for 5 min and then deposited on copper mesh. Size and morphology were observed by TEM (HT7700‐SS/FEI Tecnai G20 TWIN). Prior to SEM, the sample was fixed with glutaraldehyde, and SEM imaging was performed using a HT7700 type scanning electron microscope (Hitachi High‐Tech) with an accelerating voltage of 10 kV.

### Analysis of P5091 Loaded in P5091@RMPs‐R4F In Vitro by HPLC

Ultrasound of P5091@RMPs‐R4F was performed in methanol solution following centrifugation (14 000 *g*, 10 min). Then, the supernatants were filtered (0.2 µm filters) for HPLC (LC‐2030C Plus, designed by Shimadzu Corporation in Japan). A C18 (250 × 4.6 mm, 5 µm particle size) HPLC packed column was used as the chromatographic column. The mobile phase was CH_3_OH 0.5%TFA/H_2_O 0.5% TFA (1:1, v/v), the flow rate was 1.0 mL min^−1^, and the detection wavelength was 254 nm.

### Cell Viability

To measure cell viability, cells were plated in 96‐well plates (5000 cells per well) and allowed to grow for 24 h before treatment. Cells were then treated with different concentrations of P5091@RMPs‐R4F or other chemical reagents. After incubation for 24 h, cell viability was evaluated using a CCK‐8 assay kit (BS350B, Biosharp).

### Generation and Activation of Mouse BMDMs

Mouse bone marrow‐derived macrophages (BMDMs) were generated as described previously.^[^ ^22^ ^]^ BMDMs were cultured with 20 ng mL^−1^ IL‐4 or 20 ng mL^−1^ IL‐13, to be polarized into IL‐4‐induced M2Φ. All cytokines were purchased from PeproTech (Rocky Hill, NJ, USA).

### In Vitro Cellular Uptake Assay

To determine the cellular co‐localization of P5091@RMPs‐R4F, M1Φ, M2Φ, LLC cells, or BV2 cells were seeded in a glass‐bottom cell culture dish (NEST, catalog no. 801001; 1 × 10^5^ per well) and incubated with DiD‐labeled RMPs for 3 h. Subsequently, these cells were washed three times in PBS and then stained with phalloidin (10 µm) for 10 min. After that, cells were washed with PBS and fixed in 4% paraformaldehyde for 30 min and then washed in PBS again. Cells were imaged by confocal laser scanning microscopy (LSM 710). For quantitative assessment of cellular uptake, cells were seeded in six‐well cell culture dishes and treated as above, then washed in PBS three times, collected, fixed, and resuspended in PBS (150 *µ*L) for flow cytometry detection.

### Native SDS‐PAGE to Verify the P5091@RMPs‐R4F Stability

Operation was similar to conventional SDS‐PAGE, except the gel and electrophoresis buffers did not contain SDS. P5091@RMPs‐R4F was incubated with mouse serum or fetal bovine serum for different time, and then a certain amount of glycerol was added for convenient sampling. Fluorescence intensity of CFSE (5(6)‐CFDA *N*‐succinmidyl ester) and DIR were obtained using an ultra‐sensitive exposure instrument (BIO‐RAD, ChemiDoc MP Imaging System), and images were processed using Image J.

### Western Blotting

Cells or RMPs were treated with RIPA lysis buffer and protein separation was performed on 8% SDS‐PAGE gel, followed by membrane transfer (0.2 µm PVDF membrane, Millipore) and antibody incubation. SR‐B1 antibody (Catalog number: 21277‐1‐AP), TSG101 antibody (Catalog number: 67381‐1‐Ig), CD9 antibody (Catalog number: 20597‐1‐AP), Phospho‐JNK (Tyr185) Recombinant antibody (Catalog number: 80024‐1‐RR), Phospho‐ERK1/2 (Thr202/Tyr204) Polyclonal antibody (Catalog number: 28733‐1‐AP), and Phospho‐p38 MAPK (Thr180/Tyr182) Polyclonal antibody (Catalog number: 28796‐1‐AP) were purchased from Proteintech. All images were acquired using the ChemiDoc Imaging System (Bio‐Rad).

### Cytokine Detection

Blood was collected from the orbital venous plexus, and serum was collected for cytokine detection after blood coagulation at room temperature for 4 h. The LEGENDplex Mouse Cytokine Release Syndrome Panel (13‐plex) with VBottom Plate (purchased from Biolegend) was used for cytokine detection.

### Transwell Experiment

Transwell (3 µm diameter, Invitrogen, Eugene, OR, USA) inserts were infused with 0.3% gelatin (w/v) (Sigma‐Aldrich) for 24 h followed by the transfer of 5 × 10^3^ bEnd.3 cells (purchased from ATCC, Manassas, VA, USA). At the same time, M2 BMDM, BV2, and LLC cells were cultured in the lower insert of the Transwell. After culturing in the insert for 3 d, RMPs, RMPs‐R4F, or P5091@RMPs‐R4F stained with PKH26 were added to the upper insert and the culture was incubated in 5% CO_2_ at 37 °C for 24 h, followed by quantitative assessment of cellular uptake in the lower inset through flow cytometry.

### Animal Model Experiments and Evaluation of Therapeutic Effects

To establish the BRM model, mice were anesthetized by administration of 1% pentobarbital sodium before all operations. LLC‐luciferase (LUC) cells (500 000 cells suspended in 25 µL of PBS) were stereotactically injected into the striatum of the right ventricle. Four days after inoculation with LLC‐LUC cells, each mouse was observed by bioluminescence imaging to ensure that LLC brain metastasis had been established successfully and uniformly. Mice were then randomized to four groups, including control, P5091, RMPs‐R4F, and P5091@RMPs‐R4F, and given the respective treatment. Mice were treated with *i.v*. injections of 100 µL liquid four times at 2‐day intervals, or by intraperitoneal injection with P5091 (equivalent to P5091@RMPs‐R4F), four times at 2‐day intervals. To evaluate LLC brain metastasis, six mice in each group were imaged on the day when all treatments were completed under 1% pentobarbital sodium anesthesia using the Bruker In Vivo MS FX PRO Imager. To calculate the survival rate of mice in different treatment groups, after establishing the BRM model, treatment was administered on the 5th, 7th, 9th, 13th, and 15th days, and PD‐1 antibody (BioXell, Cat number: BE0146‐100 mg, 20 mg kg^−1^) was injected intraperitoneally on the 8th, 11th, and 14th days.

### Fluorescence and Bioluminescence Imaging

After Lewis brain metastasis mice had been anesthetized with 1% pentobarbital sodium, they were intraperitoneally injected with firefly luciferin (150 mg kg^−1^; Sigma‐Aldrich; CAS: 103404‐75‐7). After 15 min, mice were imaged using the Bruker In Vivo MS FX PRO Imager. The luminescent images were acquired with 3‐min exposure times, and X‐ray photographs were taken with 30 s exposure times. To image the distribution of RMPs, RMPs‐R4F, or P5091@RMPs‐R4F stained with DiD (purchased from Beyotime), the 540/20 nm excitation filter and 620/20 nm emission filter were used and the exposure time was 15 s.

### Tissue Multicolor Immunofluorescent Staining

Tissue multicolor immunofluorescent staining was performed using the OpalTM 7‐Color Manual IHC Kit (NEL811001KT, Perkinelmer). Tumor tissues were fixed and embedded in paraffin and sectioned with a microtome. The sections were routinely dewaxed and hydrated. Tris‐EDTA Buffer solution was applied for antigen retrieval, 3% H_2_O_2_ was used to quench endogenous peroxidases, and samples were blocked in normal goat serum. The slides were then incubated with Brilliant Violet 421 anti‐mouse CD206 (MMR) Antibody (Biolegend, cat# 141717) and Alexa Fluor 488 anti‐mouse F4/80 Antibody (Biolegend, cat# 123119) overnight. Next, they were incubated with DAPI for 1 h at room temperature. Finally, tissue immunofluorescence was analyzed using the PE Vectra (Perkinelmer).

### Collection of Tumor‐Infiltrating Immunocytes

Tumor‐infiltrating immune cells were obtained from BRM tissue as previously described.^[^ ^24^ ^]^

### TAM Sorting and RNA Sequencing

TAMs were sorted from tumor tissues of mice treated with either vehicle or P5091@RMPs‐R4F by flow cytometry. The sorted TAMs were washed twice with PBS, centrifuged for 10 min at 1000 *g*, and the supernatant was discarded. The cell pellet was quickly frozen at −80 °C. Then, TAMs were sent to Beijing Novogene Technology Co., Ltd for RNA sequencing.

### Histopathology and TUNEL Assay

For CNS histopathological assessment and tumor apoptosis detection, mice were transcardially perfused with PBS and then with 4% paraformaldehyde. Part of each tissue sample was treated with ethanol and xylene, and paraffin‐embedded. Sections of 5 µm thickness were stained with Luxol fast blue (LFB). Another part of each tissue sample was stained with the TUNEL probe. Images were acquired using the Nikon Ni‐E microscope (Nikon, Tokyo, Japan).

### Flow Cytometry

For cell‐surface analysis, cells were stained with the anti‐mouse Zombie NIR Fixable Viability Kit (423106), and incubated with antibodies against CD45 (103114), CD11b (101205), F4/80 (123121), CD3 (100212), CD4 (100408), and CD8a (100752) at the recommended concentrations at 4 °C for 30 min. For the T‐cell intracellular IFN‐*γ* (505808) cytokine staining, cells were fixed and permeabilized after stimulation with Phorbol 12‐myristate 13‐acetate (PMA) (ab120297, Abcam, 100 ng mL^−1^), Monensin sodium salt (ab120499, Abcam, 1 ug mL^−1^), and Ionomycin calcium salt (5608212, PeproTech, 100 ng mL^−1^) for 6 h. For the CD206 (141706) staining, cells were also fixed and permeabilized. All flow cytometry antibodies were purchased from Biolegend (San Diego, CA, USA).

### Statistical Analysis

The unpaired two‐tailed Student's *t*‐test to compare the differences between two groups was used, while survival rates were evaluated with the log‐rank Mantel‐Cox test using Graphpad Prism 7 software. Repeated measurements of tumor volume growth were compared using One‐way analysis of variance (ANOVA). Flow cytometry data were analyzed using FlowJo.10. Significant differences between the groups are indicated by *p < 0.05, **p < 0.01, and ***p < 0.001.

## Conflict of Interest

The authors declare no conflict of interest.

## Data Statement

Sample sizes were predetermined based on previous experience using at minimum three groups of mice, and all experiments were replicated at least twice to confirm findings. Statistical analyses were conducted with a two‐tailed unpaired *t*‐test or one‐way ANOVA as described below. Mice were randomly assigned to treatment groups, and where possible, treatment groups were blinded until statistical analysis. No animals or potential outliers were excluded from the data sets presented in this study. The data used and analyzed during the current study are available from the corresponding author on reasonable request.

## Supporting information

Supporting Information

## Data Availability

The data that support the findings of this study are available from the corresponding author upon reasonable request.
