# Supplementary material for: Engineered Microparticles for Treatment of Murine Brain Metastasis by Reprograming Tumor Microenvironment and Inhibiting MAPK Pathway
Source: Adv Sci (Weinh). 2023 Jan 25;10(8):2206212. doi: 10.1002/advs.202206212 (PMC10015898; doi:10.1002/advs.202206212)
Supplement: Supplementary file 1 — Supporting Information [file ADVS-10-2206212-s001.pdf]

ADVANCED SCIENCE
Open Access

Supporting Information

for *Adv. Sci.*, DOI 10.1002/advs.202206212

Engineered Microparticles for Treatment of Murine Brain Metastasis by Reprograming Tumor Microenvironment and Inhibiting MAPK Pathway

Lisen Lu, Huaduan Zi, Jie Zhou, Jing Huang, Zihan Deng, Zijian Tang, Li Li, Xiujuan Shi, Pui-Chi Lo, Jonathan F. Lovell, Deqiang Deng, Chao Wan* and Honglin Jin**

Supplementary information

Engineered microparticles for treatment of murine brain metastasis by reprograming tumor microenvironment and inhibiting MAPK pathway

Lisen Lu[a,b,1], Huaduan Zi[c,1], Jie Zhou [b,1], Jing Huang[a], Zihan Deng[a], Zijian Tang[a], Li Li[a], Xiujuan Shi[a], Pui-Chi Lo[d], Jonathan F. Lovell[d], Deqiang Deng[a,*], Chao Wan [b,*], Honglin Jin[a,*]

[a]College of Biomedicine and Health and College of Life Science and Technology, Huazhong Agricultural University, Wuhan, 430070, China

[b]Cancer Center, Union Hospital, Tongji Medical College, Huazhong University of Science and Technology, Wuhan 430022, China

[c]Beijing Institute of Clinical Research, Beijing Friendship Hospital, Capital Medical University, Beijing 100050, China.

[d]Department of Biomedical Sciences, City University of Hong Kong, Tat Chee Avenue, Kowloon, Hong Kong, China

[e]Department of Biomedical Engineering, University at Buffalo, State University of New York, Buffalo, NY 14260, USA

[1] These authors contributed equally to this work.

[*]Corresponding authors.

E-mail addresses: jin@hust.edu.cn (H. Jin), wanc@hust.edu.cn (C. Wan).

KEYWORDS. Cancer immunotherapy, Extracellular vesicles, Macrophages polarization, Blood brain barrier, USP7

Table 1. Protein concentration of obtained MPs treated in different ways.

Cell processing	Concentration (mg/mL)
PBS	0.12 ± 0.02
DDP	0.26 ± 0.05
UV	1.13 ± 0.09
RT	1.12 ± 0.08

Table 2. The sequence of target gene.

Gene name	Sequence
R4F-GFP-GPI	GCCACCATGAGGCTCACCGTGGGTGCCCTGCTGGCCTGCG<br>CTGCCCTGGGGCTGTGTCTGGCTGGTGGGTCAGGTGGGAG<br>CTTCGCCGAAAAGTTCAAAGAGGCTGTGAAGGATTACTTC<br>GCTAAGTTCTGGGACGGTGGGTCAGGTGGGAGCATGGTGA<br>GCAAGGGCGAGGAGCTGTTCACCGGGGTGGTGCCCATCCT<br>GGTCGAGCTGGACGGCGACGTAAACGGCCACAAGTTCAG<br>CGTGTCCGGCGAGGGCGAGGGCGATGCCACCTACGGCAA<br>GCTGACCCTGAAGTTCATCTGCACCACCGGCAAGCTGCCC<br>GTGCCCTGGCCCACCCTCGTGACCACCCTGACCTACGGCG<br>TGCAGTGCTTCAGCCGCTACCCCGACCACATGAAGCAGCA<br>CGACTTCTTCAAGTCCGCCATGCCCGAAGGCTACGTCCAG<br>GAGCGCACCATCTTCTTCAAGGACGACGGCAACTACAAGA<br>CCCGCGCCGAGGTGAAGTTCGAGGGCGACACCCTGGTGAA<br>CCGCATCGAGCTGAAGGGCATCGACTTCAAGGAGGACGG<br>CAACATCCTGGGGCACAAGCTGGAGTACAACTACAACAGC<br>CACAACGTCTATATCATGGCCGACAAGCAGAAGAACGGC<br>ATCAAGGTGAACTTCAAGATCCGCCACAACATCGAGGACG<br>GCAGCGTGCAGCTCGCCGACCACTACCAGCAGAACACCCC<br>CATCGGCGACGGCCCCGTGCTGCTGCCCGACAACCACTAC<br>CTGAGCACCCAGTCCGCCCTGAGCAAAGACCCCAACGAGA<br>AGCGCGATCACATGGTCCTGCTGGAGTTCGTGACCGCCGC<br>CGGGATCACTCTCGGCATGGACGAGCTGTACAAGGGTGGC<br>AGCTCCCTGCAGTCCACAGCTGGTCTCCTGGCTCTCTCTCT<br>CTCTCTTCTACATCTCTACTGT

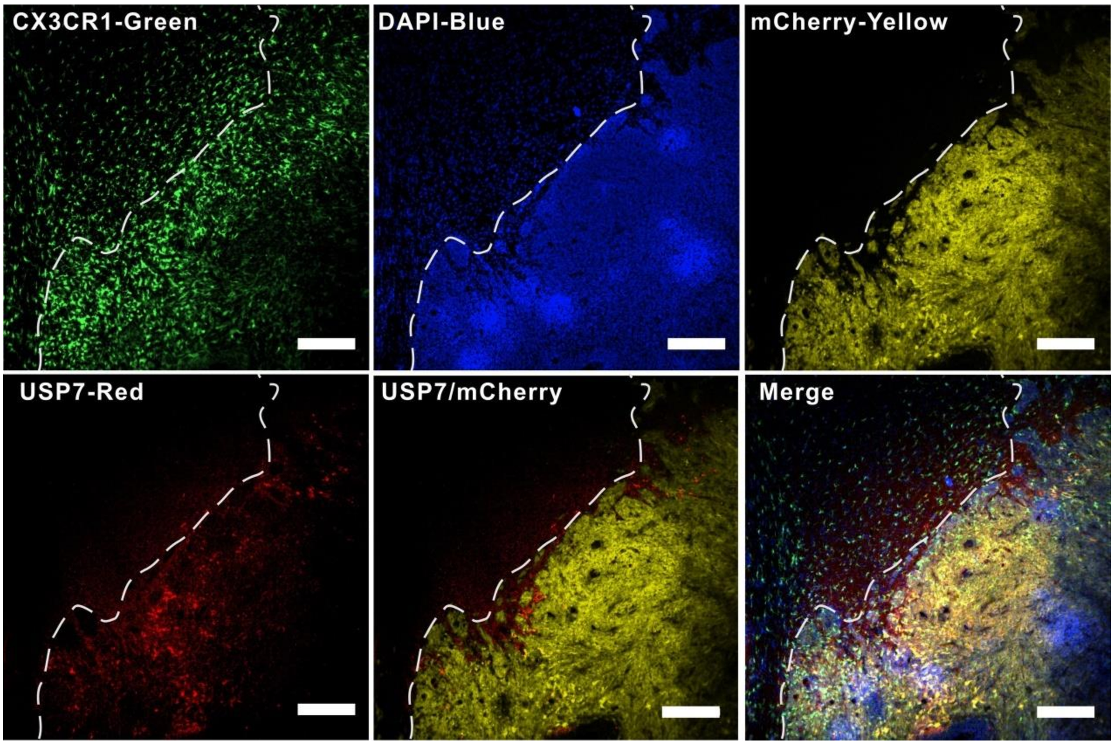

Figure S1. To verify the expression of USP7 in mouse BRM model. CX3CR1$^{GFP/+}$ transgenic mice (purchased from Jackson laboratory, Strain#: 005582) and LLC-mCherry cells were used in this model. Scale bar is 200 μm.

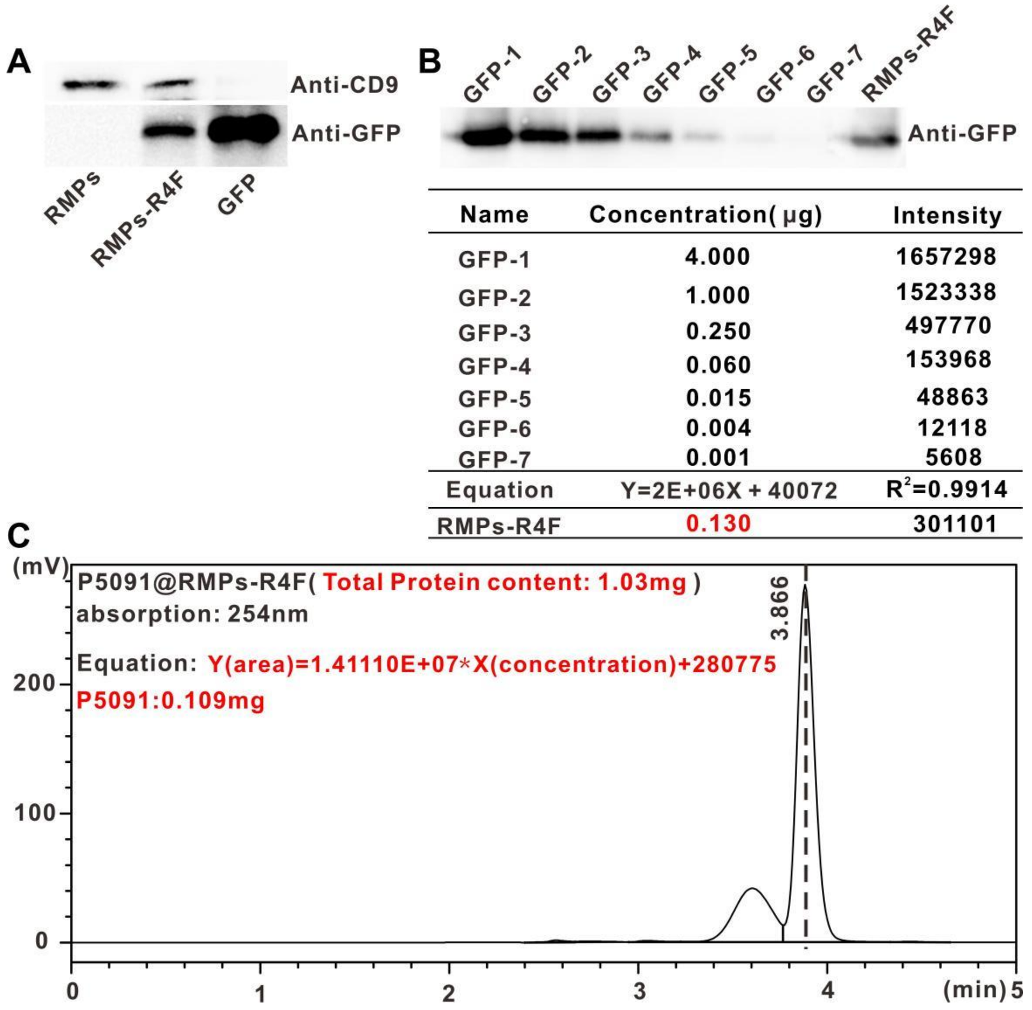

Figure S2. Identify the content of P5091 and R4F in P5091@RMPs-R4F. (A) The expression of GFP in RMPs-R4F or in RMPs. (B) Identify the gray-scale values of different concentrations of GFP protein and GFP contained in RMPs-R4F. (C) Using HPLC to determine the content of P5091 in RMPs-R4F.

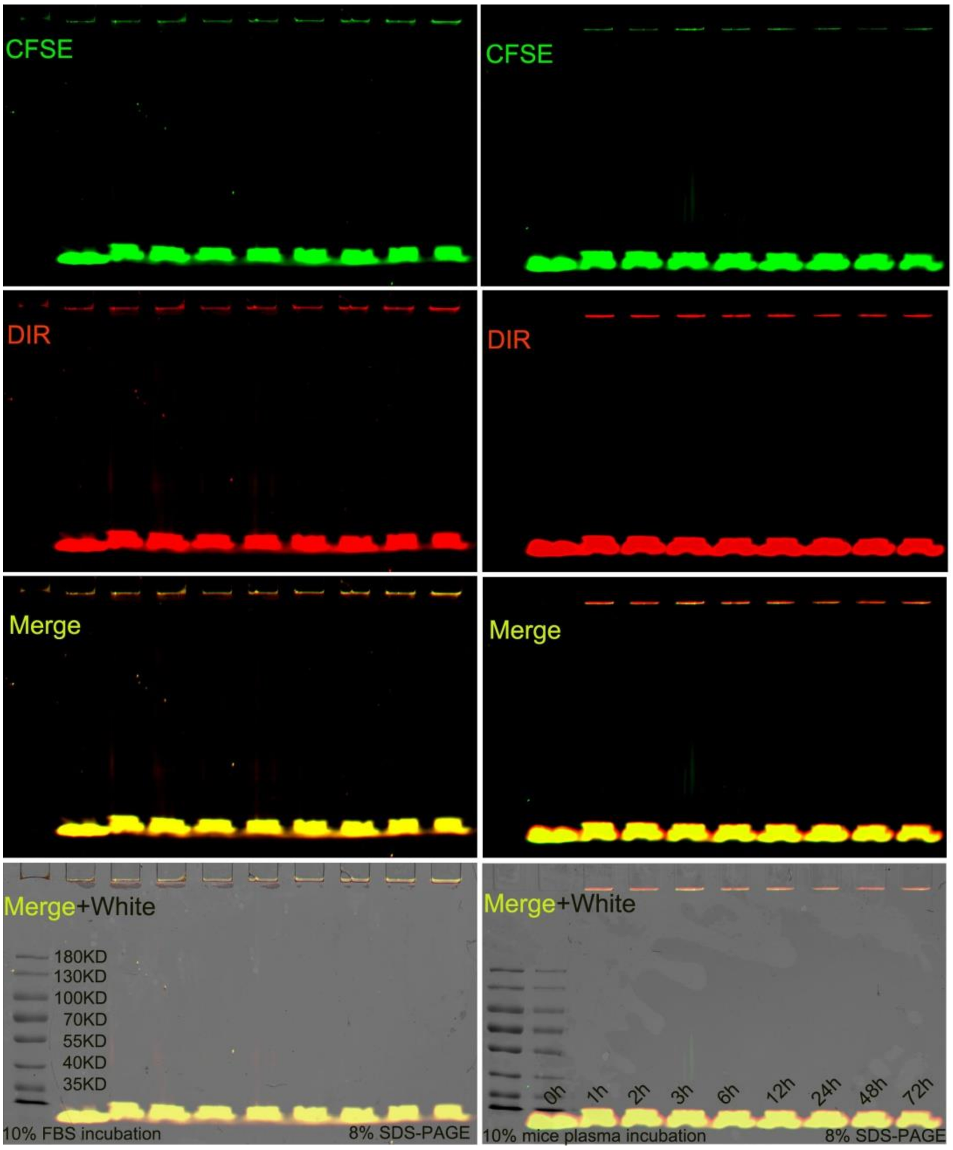

Figure S3. The stability of P5091@RMPs-R4F in mouse serum and fetal bovine serum was determined by native SDS-PAGE. Green represents CFSE dye, Red represents DIR and Yellow represents the col-location of cell membrane and cell contents.

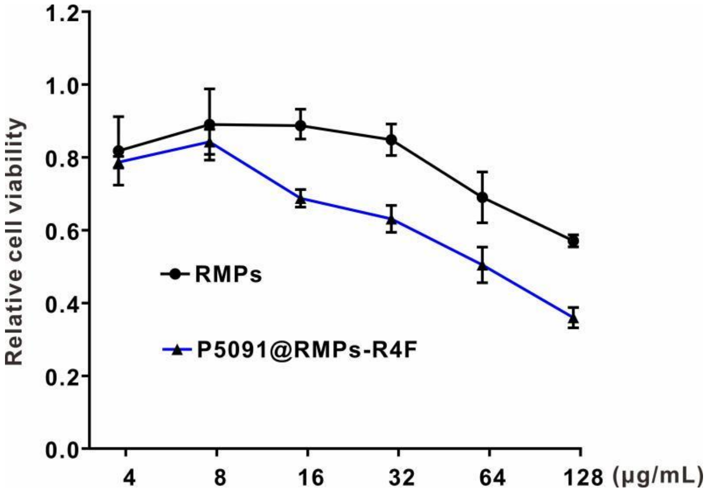

Figure S4. Assessment of the therapeutic ability of P5091@RMPs-R4F against LLC cells using CCK8 assay.

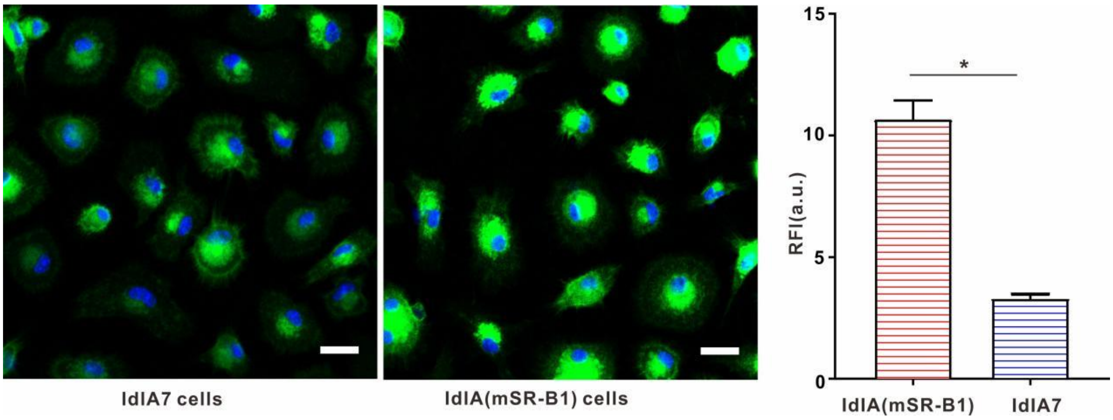

Figure S5. Analysis of the specific targeting properties of P5091@RMPs-R4F to SR-B1 receptor. Scale bar is 20 μm. Blue is DAPI, Green is PKH67. Statistical analysis was performed using unpaired t-test. The data represented as the mean ± SEM (n = 4). *P < 0.05, **P < 0.01, ***P < 0.001.

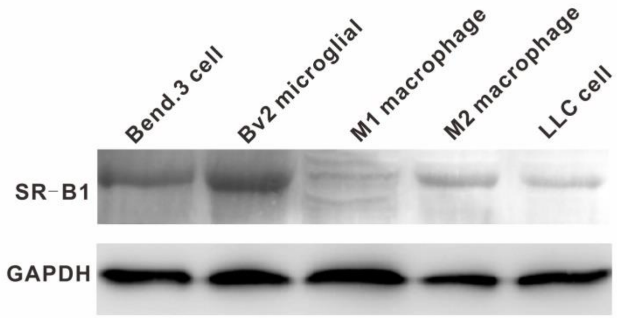

Figure S6. Analysis of the expression of SR-B1 receptor in bEnd.3, M1Φ, M2Φ, BV2 microglial and LLC cells by western blotting.

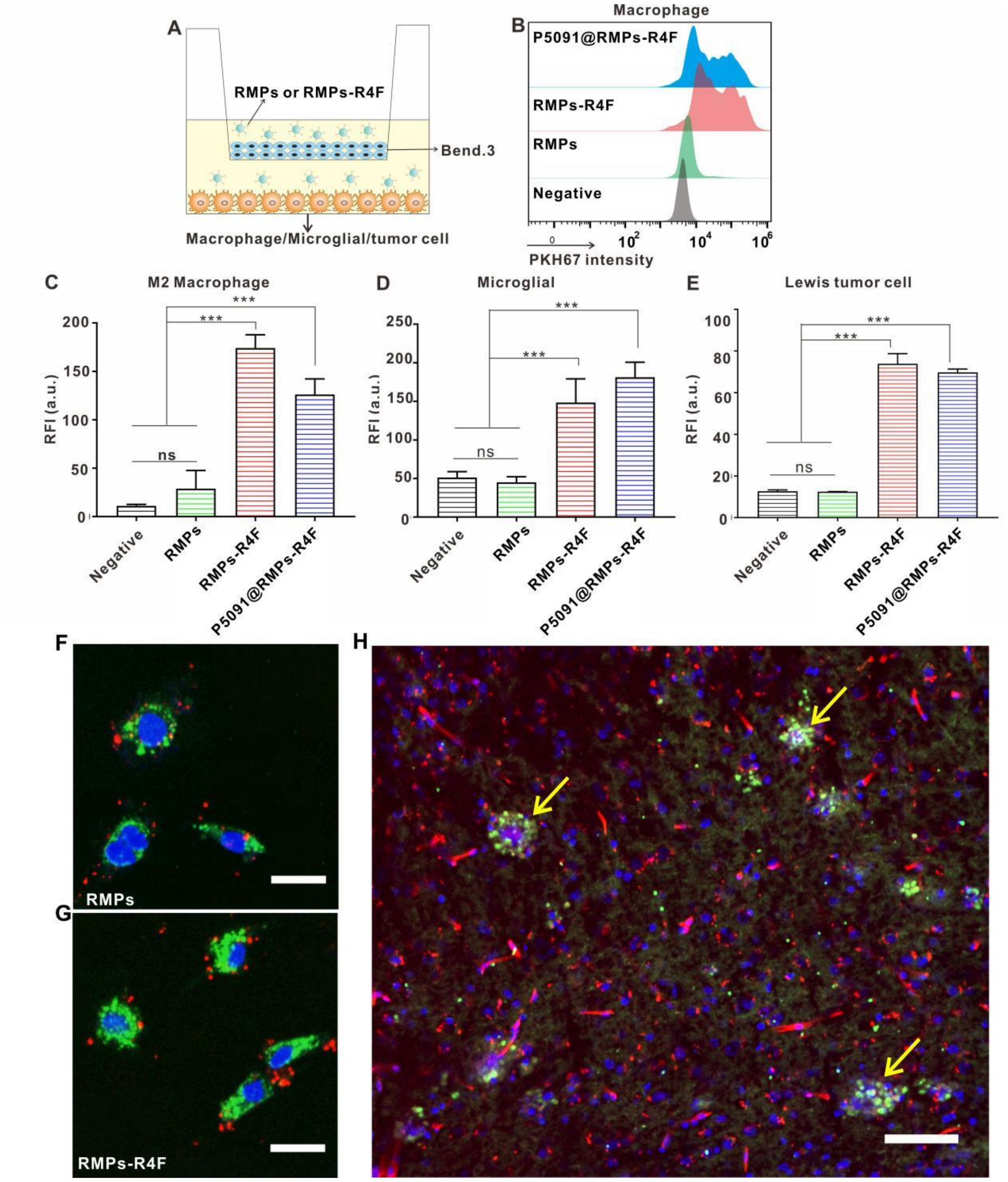

Figure S7. Transwell experiment to verify the ability of crossing BBB. (A) Schematic diagram of transwell experiment. (B) Representative fluorenscent intensity of M2Φ. The M2Φ were incubated with PKH67 stained MPs (RMPs, RMPs-R4F or P5091@RMPs-R4F) that were added in the upper chamber for 24 h and followed with flow cytometry analysis. (C-E) Statistics of fluorenscent intensity of M2Φ, BV2 microglial, LLC cells 24 h after different PKH67 stained MPs (RMPs, RMPs-R4F or P5091@RMPs-R4F) were added to the upper chamber by using flow cytometry. Statistical analysis was performed using one-way ANOVA with Tukey's multiple

comparison test. Data are presented as the mean ± SEM (n = 3). *P < 0.05, **P < 0.01, ***P < 0.001, and ns: not significant. (F-G) Confocal imaging analysis of MPs transendocytosis. Scale bar is 20 μm. (H) Immunofluorescence was used to verify the expression of SR-B1 receptor in the BBB of mice. Red-CD31, Green-SR-B1. Scale bar is 100 μm.

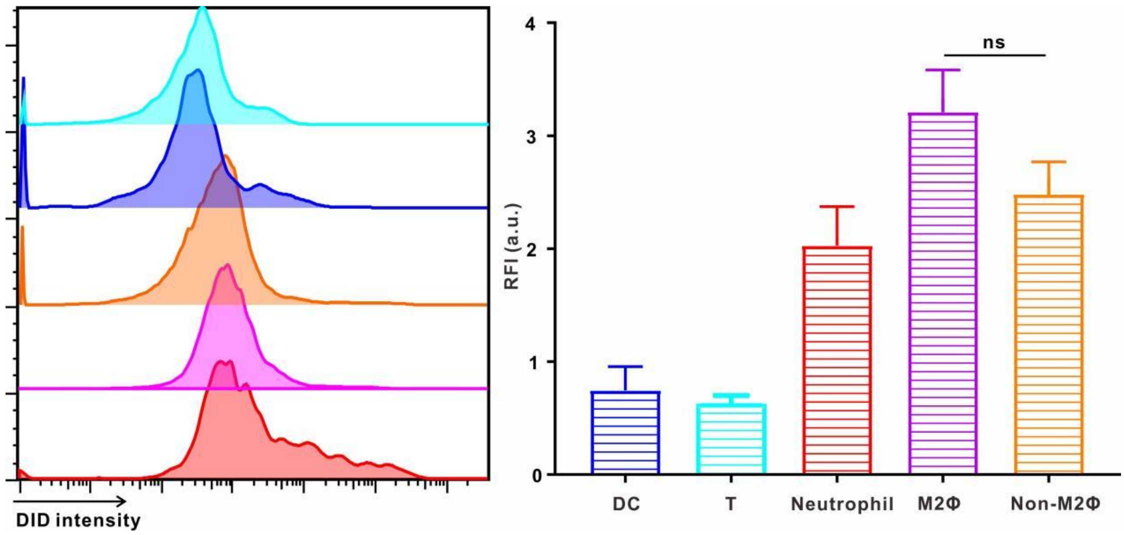

Figure S8. Calculation of the MPs uptake by infiltrating immune cells in brain tumors after *i.v.* injection of RMPs. Statistical analysis was performed using one-way ANOVA with Tukey's multiple comparison test (n = 3). Data are presented as the mean ± SEM. *P < 0.05, **P < 0.01, ***P < 0.001.

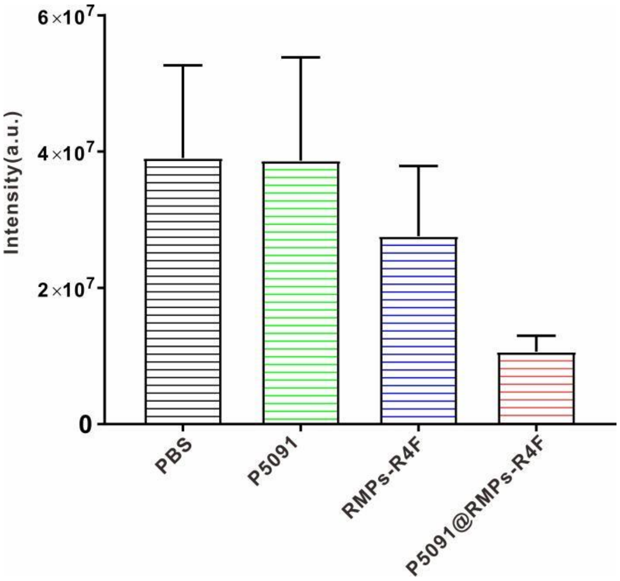

Figure S9. Statistics of luminescence intensity of brain in different treatment groups (n = 6). Data are presented as the mean ± SEM.
